# Left Ventricular Assist Device: Review of Antimicrobial Prophylaxis Strategies and Incidence of Infections at a Tertiary Care Center 12-Year Experience

**DOI:** 10.1093/ofid/ofad465

**Published:** 2023-09-08

**Authors:** Maria Alejandra Mendoza, Nischal Ranganath, Bismarck Bisono Garcia, Ryan W Stevens, Brian Lahr, John O’Horo, John Stulak, Aditya Shah

**Affiliations:** Division of Public Health, Infectious Diseases, and Occupational Medicine News, Mayo Clinic, Rochester, Minnesota, USA; Division of Public Health, Infectious Diseases, and Occupational Medicine News, Mayo Clinic, Rochester, Minnesota, USA; Division of Public Health, Infectious Diseases, and Occupational Medicine News, Mayo Clinic, Rochester, Minnesota, USA; Department of Pharmacy Services, Mayo Clinic, Rochester, Minnesota, USA; Department of Quantitative Health Sciences, Mayo Clinic, Rochester, Minnesota, USA; Division of Public Health, Infectious Diseases, and Occupational Medicine News, Mayo Clinic, Rochester, Minnesota, USA; Division of Cardiovascular Surgery, Mayo Clinic, Rochester, Minnesota, USA; Division of Public Health, Infectious Diseases, and Occupational Medicine News, Mayo Clinic, Rochester, Minnesota, USA

**Keywords:** LVAD, surgical prophylaxis

## Abstract

**Background:**

Left ventricular assist devices (LVAD) have an associated infection rate of 13%–80% postimplant. An optimal strategy for surgical infection prophylaxis (SIP) at the time of implantation has not been well defined. We aimed to evaluate the different LVAD implantation antibiotic prophylaxis regimens as well as the incidence of LVAD infection at our institution.

**Methods:**

We performed a single-center, retrospective study of patients who underwent LVAD implantation between February 2007 and June 2019. The primary outcome was the incidence of LVAD infection (LVADI), within 3 months and 1 year of placement, between patients who received expanded or narrow-spectrum regimens for SIP. We assessed outcomes using Kaplan-Meier, time-to-first event. We used a noninferiority analysis, which was established if the narrow-spectrum event rate was no more than 5% greater than the expanded-spectrum event rate.

**Results:**

We included 399 patients, 305 (76.4%) patients received narrow-spectrum SIP, whereas the remaining 94 (23.6%) patients received the expanded-spectrum regimen. Statistical noninferiority of the narrow spectrum to the multiple drug regimen was demonstrated at both time points, and statistical superiority of the narrow-spectrum group across 12-month follow up was further evident (*P* = .037).

**Conclusions:**

We report evidence supporting noninferiority, or even superiority, of the narrow-spectrum over expanded-spectrum antimicrobial prophylaxis strategy with respect to LVADI. These findings support data-driven antimicrobial prophylaxis strategies.

Congestive heart failure (CHF) is a primary cause of cardiovascular morbidity and mortality, affecting more than 64 million people worldwide [1]. In the United States, it is estimated that CHF will affect 8 million people by 2030 [2], with a subset of patients having advanced heart failure. Although medical management remains the cornerstone of therapy, patients with advanced CHF may benefit from mechanical circulatory support such as left ventricular assist device (LVAD) either as a destination therapy or a bridge to heart transplant. In 2019, more than 3000 patients underwent LVAD placement [3].

Unfortunately, mechanical circulatory therapy is associated with multiple complications, most importantly, infections. The INTERMACS registry from 2006 to 2009 reported an infection rate between 13% and 80% with 16% of post-LVAD deaths associated with infection [4]. Despite data supporting higher risk of infection, an optimal strategy for surgical infection prophylaxis (SIP) at the time of implantation has not been well defined and practices vary widely [5]. The primary challenge to developing a consensus SIP approach is that patients requiring LVAD implantation are often critically ill and have competing infectious risk factors such as longer intensive care unit (ICU) or hospital stay, higher rates of invasive accesses and procedures, and antimicrobial exposure.

Consequently, prolonged or unneeded exposure to multiple classes of antibiotics can increase healthcare costs and impacts patient outcomes through increased risk of *Clostridioides difficile* infection (CDI) [6], acute kidney injury, and emergency of antimicrobial-resistant microorganisms. We present our experience at Mayo Clinic in Rochester Minnesota where we evaluated the different LVAD implantation antibiotic prophylaxis strategies as well as the incidence of LVAD infection (LVADI).

## METHODS

### Patient Consent

Patients included in this study have provided research authorization for the confidential clinical use of information to Mayo Clinic. The design of the work has been approved by local ethical committee at Mayo Clinic. The Institutional Review Board number is 20-005814.

We performed a single-center, retrospective study of patients who underwent LVAD implantation between February 2007 and June 2019. Our primary outcome was the incidence of LVADI, within 3 months and 1 year of placement. Secondary outcomes included 3-month and 12-month all-cause mortality after LVAD placement as well as incidence of CDI.

We defined LVADI according to the International Society of Heart and Lung transplantation (ISHLT) criteria, classifying infections into VAD-specific, VAD-related, and non-VAD-related [7]. For the VAD-specific infections, we collected information on pump, cannula, pocket, and driveline infections. For the VAD-related infections, we collected episodes of bloodstream infections (BSIs), endocarditis, and mediastinitis. We did not collect information on non-VAD-related infections, such as pneumonia or urinary tract infections.

For SIP, we categorized the antibiotic regimens as narrow or expanded spectrum. We included regimens started only for prophylaxis; we excluded antibiotic regimens used as therapy for infectious conditions at the time of LVAD placement. We defined narrow-spectrum SIP as cefazolin only, vancomycin only, or cefazolin and vancomycin. We defined expanded-spectrum SIP as any of the narrow-spectrum regimens plus another antimicrobial, either antibacterial or antifungal. We included data of microbiologically proven infections only, and the date of the infection was defined as the date on which the first positive culture was collected. We also collected other variables such as the following: Charlson comorbidity index, Acute Physiologic Assessment and Chronic Health Evaluation (APACHE) III score, and Sequential Organ Failure Assessment (SOFA) score in patients who were in the ICU before LVAD placement, length of intubation during and around LVAD placement admission, and central or arterial lines and urinary catheters at the time of LVAD placement.

### Statistical Analysis

Demographic and clinical characteristics are summarized with medians and interquartile ranges (IQR) for continuous variables and percentages for categorical variables. The percentages for events were computed using time-to-event (Kaplan-Meier) methods that account for some losses during the follow-up period. We compared each of these factors between narrow and expanded-spectrum SIP groups using Wilcoxon tests for continuous variables and Pearson χ^2^ tests for categorical variables. Additional comparisons were made on variables serving as a surrogate for severity of illness (eg, hospital length of stay) or risk factors for LVAD infection (eg, central line). We assessed outcomes using Kaplan-Meier time-to-first-event analyses to account for patients lost to follow up. Cumulative event rates for each outcome (LVAD infection, CDI, and death) over time since LVAD placement were estimated using Kaplan-Meier time-to-first-event analyses to account for patients lost to follow up. For both LVAD infection and CDI outcomes, the noninferiority of the narrow-spectrum group to the expanded-spectrum group was established whether the narrow-spectrum event rate was no more than 5% greater than the expanded-spectrum event rate; a 5% margin was clinically considered noninferior by the investigators. This was established whether the upper boundary of the 2-sided 95% confidence interval (CI) of the difference in rates of patients in the narrow- versus extended-spectrum drug group, estimated separately at 3-, 6-, and 12-month follow up, was less than +5.0%. When noninferiority was demonstrated, we assessed superiority of the narrow-spectrum group to the expanded-spectrum group cumulatively over the 12-month follow up using a Mantel-Haenszel log-rank test. Finally, to assess the long-term risk of death from an intervening LVAD infection and relevant covariates, we conducted a multivariable Cox regression analysis with LVAD infection incorporated in the model as a time-dependent variable. We performed all analyses using R statistical software (version 4.1.2; R Foundation for Statistical Computing, Vienna, Austria).

## RESULTS

We included 399 patients; the majority (316; 79.2%) were male with a median age of 62.4 (IQR, 53.2–69.2) years. The median Charlson comorbidity index score was 6 (IQR, 4–8). Overall, 156 (39.1%) had a LVAD placed as a bridge to therapy, whereas the remainder, at least initially, had a LVAD placed as destination therapy. Before implantation of LVAD, 35 (8.8%) patients tested positive for methicillin-resistant *Staphylococcus aureus* (MRSA) colonization, whereas 38 (9.5%) tested positive for vancomycin-resistant enterococci. The most common type of LVAD implanted was HeartMate-2, in 294 (73.7%) cases, followed by HeartWare Ventricular Assist Device (HVAD) in 75 (18.8%) cases.

Overall, 305 (76.4%) patients received narrow-spectrum-SIP, whereas the remaining 94 (23.6%) patients received an expanded-spectrum regimen. The most common medication in the narrow-spectrum regimen was cefazolin only (n = 241, 79%) followed by cefazolin plus vancomycin (n = 135, 44.3%). Twenty-seven patients received vancomycin alone (8.9%). In the expanded-spectrum cohort, the agent most commonly added to narrow-spectrum drug regimens was cefepime (48.9%). For antifungal coverage, 25.5% (24 of 94) of patients received an antifungal such as fluconazole or echinocandin. Rifampin was used in 7.4% (7 of 94) of patients and was more commonly encountered in the earlier years of our cohort, owing to practice evolution in the later years.

A comparison of the baseline characteristics (Table 1) showed no significant differences between the 2 prophylaxis groups, with the exception of LVAD as a bridge therapy to heart transplant, which was more common in the multidrug group (50.0% vs 35.7%, *P* = .013). At the time of ICU admission, the median SOFA and APACHE III score was 6 (IQR, 3–10) and 66 (IQR, 53–82), respectively, with no differences between groups. The median hospital stay for patients in the narrow-spectrum group was 27.8 days compared with 28.3 days for those in the expanded-spectrum-SIP group (*P* = .253), whereas the median ICU stay was 9 days in the narrow-spectrum-SIP group versus 8.9 days in the expanded-spectrum group (*P* = .544).

**Table 1.** Baseline Characteristics by Antimicrobial Prophylaxis Group

Characteristic	N	Narrow Spectrum (N = 305)	Expanded Spectrum (N = 94)	*P* Value
Age at time of LVAD, years	399	62.4 (53.4–69.3)	62.3 (52.0–67.0)	.655^a^
Male gender	399	244 (80.0%)	72 (76.6%)	.477^b^
Year of LVAD	399	2013 (2010–2015)	2014 (2010–2016)	.089^a^
Indication for LVAD Bridge	399	109 (35.7%)	47 (50.0%)	.013^b^
MRSA colonization	399	23 (7.5%)	12 (12.8%)	.117^b^
VRE colonization	399	25 (8.2%)	13 (13.8%)	.104^b^
Type of LVAD	399	…	…	.515^b^
HM2	…	229 (75.1%)	65 (69.1%)	
HVAD	…	54 (17.7%)	21 (22.3%)	
Other	…	22 (7.2%)	8 (8.5%)	
SOFA	345	6 (3–9)	6 (2–10)	.762^a^
APACHE III score	345	66 (52–83)	69 (56–81)	.483^a^
Charlson comorbidity index	399	6 (5–8)	6 (4–8)	.528^a^

Abbreviations: APACHE, Acute Physiologic Assessment and Chronic Health Evaluation; HM2, Heartmate 2; HVAD, HeartWare Left Ventricular Assist Devices; ICU, intensive care unit; LOS, length of stay; LVAD, left ventricular assistant device; MRSA, methicillin-resistant *Staphylococcus aureus*; SOFA, Sequential Organ Failure Assessment; VRE, vancomycin-resistant enterococci.

Note: Values represent median, lower quartile, and upper quartile for continuous variables, whereas frequencies and percentages are presented for categorical variables. N is the number of nonmissing values.

a Wilcoxon 2-sample test.

b Pearson χ² test.

Surrogate measures for severity of illness or risk factors for LVADI were also assessed for group differences. All patients underwent mechanical ventilation due to the surgery for a median duration of 1 day (IQR, 1–2), and although there were some rare cases intubated from several days up to 90 days, there was no difference in ventilation days between groups (*P* = .54). Central lines were placed in 85.7% of patients for a median duration of 5 days, whereas 53.6% of patients had an arterial line for a median duration of 3 days. There were no statistically significant differences between groups in terms of line usage or duration. Urinary catheter was present in 12.6% of patients, with no group difference observed (*P* = .672) (Table 2).

**Table 2.** Surrogate Measures of Disease Severity by Antimicrobial Prophylaxis Group

Measure	N	Narrow Spectrum (N = 305)	Expanded Spectrum (N = 94)	*P* Value
ICU length of stay, days	399	9.0 (5.9–16.9)	8.9 (5.9–23.1)	.544^a^
Hospital length of stay, days	399	27.8 (18.1–42.9)	28.3 (18.9–46.0)	.253^a^
Hospital mortality	399	26 (8.5%)	10 (10.6%)	.532^b^
Central line	399	262 (85.9%)	80 (85.1%)	.847^b^
D with central line	342	5.0 (3.0–11.0)	6.0 (3.0–10.0)	.878^c^
Arterial line	399	162 (53.1%)	52 (55.3%)	.708^b^
D with arterial line	214	3.0 (3.0–5.0)	4.0 (3.0–7.0)	.072^c^
Foley catheter	398	37 (12.2%)	13 (13.8%)	.672^b^

Abbreviations: ICU, intensive care unit; N, number of nonmissing values.

Note: Values represent median, lower quartile, and upper quartile for continuous variables, whereas frequencies and percentages are presented for categorical variables.

a Mantel-Haenszel log-rank test.

b Pearson χ² test.

c Wilcoxon 2-sample test.

### Left Ventricular Assist Device Infections

In total, 49 patients developed LVADI within 1 year of LVAD placement (Table 4). The median time to LVADI was 108 days (IQR, 41–175). The most common types of LVADI were bloodstream and percutaneous driveline infections in 28 (57.1%) and in 21 (42.9%) cases, respectively. The most common organisms were Gram-positive, including methicillin-susceptible *S aureus* (MSSA) (n = 13), MRSA (n = 7), *Enterococcus faecalis* (n = 5), and coagulase-negative staphylococci (n = 4). Among Gram-negative organisms, the most common was *Klebsiella* spp (n = 2). We also observed 7 polymicrobial infections, as well as 1 fungal pocket infection with *Candida glabrata*.

### Outcomes

**Figure 1.**
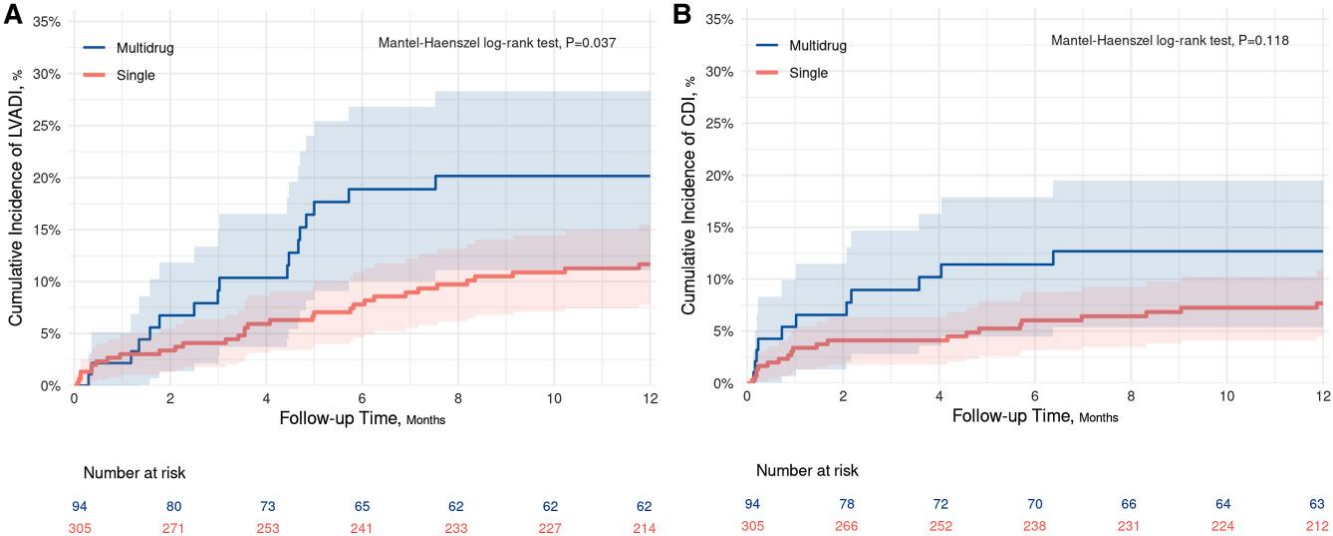
Cumulative incidence of (*A*) left ventricular assistant device (LVAD) infection and (*B*) *Clostridioides difficile*. CDI, *C difficile* infection.

**Figure 2.**
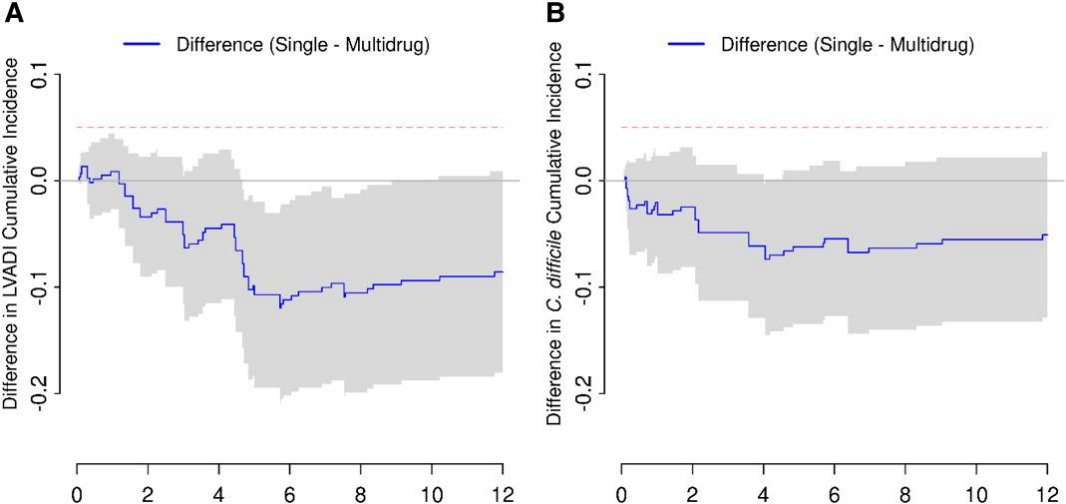
Noninferiority of narrow-spectrum (single) versus extended-spectrum (multidrug) in (*A*) left ventricular assistant device (LVAD) infection and (*B*) *Clostridioides difficile* infection.

The cumulative rates of LVADI for patients in the narrow-spectrum cohort at 3 and 12 months post-LVAD placement were 4.1% and 11.7%, respectively, whereas in the expanded-spectrum-SIP patients the corresponding rates were 9.2% and 20.3% (Figure 1*A*). The differences in 3- and 12-month cumulative incidence for the narrow- versus expanded-spectrum cohorts were −5.1% (95% CI, −11.6% to +1.4%) and −8.6% (95% CI, −18.0% to +0.9%), respectively (Figure 2*A*). Therefore, we demonstrated statistical noninferiority of the narrow-spectrum to the multiple drug regimen at both time points, and statistical superiority of the narrow-spectrum group across 12-month follow-up was further evident (*P* = .037). For the secondary outcome of CDI, the 12-month cumulative incidence was 7.7% for patients who received narrow-spectrum versus 12.8% for patients who received multidrug SIP, demonstrating noninferiority (cumulative incidence difference, −4.9% [95% CI, −11.3% to +1.5%]) but not superiority (*P* = .118) of the narrow-spectrum group. There was no difference in all-cause mortality between narrow- and extended-spectrum drug groups during the 12 months after LVAD placement (*P* = .856) (Table 3).

**Table 3.** Clinical Outcomes by Group

Outcome	Narrow Spectrum (N = 305)	Multidrug (N = 94)	Narrow-Expanded Spectrum Difference (95% Confidence Interval)	*P* Value^a^
LVAD Infection	…	…	…	.037
m = 3	12 (4.1%)	8 (9.2%)	−5.1% (−11.6% to +1.4%)	…
m = 6	22 (7.8%)	16 (19.0%)	−11.2% (−20.2% to −2.2%)	…
m = 12	32 (11.7%)	17 (20.3%)	−8.6% (−18.0% to +.9%)	…
*Clostridioides difficile* Infection	…	…	…	.118
m = 3	12 (4.1%)	8 (9.0%)	−4.9% (−11.3% to +1.5%)	…
m = 6	17 (6.0%)	10 (11.5%)	−5.5% (−12.7% to +1.8%)	…
m = 12	21 (7.7%)	11 (12.8%)	−5.1% (−12.9% to +2.7%)	…
All-Cause Mortality	…	…	…	.856
m = 3	31 (10.2%)	13 (13.9%)	−3.7% (−11.5% to +4.1%)	…
m = 6	43 (14.1%)	15 (16.0%)	−1.9% (−10.4% to +6.5%)	…
m = 12	64 (21.1%)	20 (21.4%)	−.4% (−9.9% to +9.1%)	…

Abbreviations: LVAD, left ventricular assistant device.

Note: Values represent the cumulative number of events (Kaplan-Meier cumulative incidence estimate, %) at the corresponding time point of follow up.

a Test for statistical superiority of narrow-spectrum group across 12-month follow up based on Mantel-Haenszel log-rank test.

**Table 4.** LVADI Within 1 Year of Implantation

Age	Gender	Type of LVAD	Type of SIP	Implant to Infection (days)	Type of Infection	Organism
69	M	HM2	Narrow spectrum	2	BSI	PSA
61	M	HVAD	Narrow spectrum	3	BSI	*Enterococcus faecalis*
73	M	HM2	Narrow spectrum	4	BSI	*Morganella* spp
67	M	HVAD	Narrow spectrum	4	BSI, DL	*Klebsiella pneumoniae*
80	M	HM3	Expanded spectrum	9	BSI	*Klebsiella* spp
55	M	HM2	Narrow spectrum	11	BSI	CONS
42	M	HVAD	Expanded spectrum	11	BSI, DL	*Achromobacter* species, VRE, *Cutibacterium acnes*
21	F	HVAD	Narrow spectrum	11	PCI, DL	MRSE
42	F	HVAD	Narrow spectrum	14	PCI, DL	*Staphylococcus epidermidis*
51	F	HVAD	Narrow spectrum	21	BSI, DL	*E faecalis*
49	M	HA-5	Narrow spectrum	29	DL	MRSA
44	F	HVAD	Expanded spectrum	36	BSI	*Candida glabrata*
67	M	HM2	Expanded spectrum	41	BSI, PCI	MSSA
48	M	HVAD	Expanded spectrum	48	BSI	*Escherichia coli*
59	M	HM2	Expanded spectrum	54	MN	MRSA
75	M	HM3	Narrow spectrum	54	BSI	MRSA
70	F	HM2	Narrow spectrum	64	PK	CONS
58	M	HM2	Narrow spectrum	69	PCI, PK, MN	*Rhizopus* spp, MRSE
46	M	HVAD	Expanded spectrum	76	BSI	*Enterobacter cloacae*
33	F	HVAD	Expanded spectrum	91	BSI	*Streptococcus mitis*
26	M	HM2	Expanded spectrum	92	BSI	MSSA
57	M	HVAD	Narrow spectrum	96	BSI, PCI, PK, MN	MSSA
46	M	HVAD	Narrow spectrum	104	BSI, DL, IE	MRSA
74	M	HM3	Narrow spectrum	108	BSI	MRSE
41	F	HVAD	Narrow spectrum	108	DL	MSSA
46	M	HM2	Narrow spectrum	110	DL	*E faecalis*
74	M	HM2	Narrow spectrum	124	DL	MSSA
61	M	HM2	Expanded spectrum	135	BSI, PCI, PK, DL	MSSA
47	1	HVAD	Expanded spectrum	136	BSI	*E faecalis*
57	F	HM2	Expanded spectrum	142	DL	PSA
72	F	HVAD	Expanded spectrum	143	BSI	*Staphylococcus devriesei* and *S mitis*
19	F	HM2	Expanded spectrum	147	IE	*E faecalis*
45	M	HM2	Narrow spectrum	151	PK	*C acnes*
74	M	HM3	Expanded spectrum	152	BSI	*Corynebacterium striatum*
50	F	HVAD	Narrow spectrum	152	DL	CONS, *Serratia* spp, *Acinetobacter*
61	M	HM2	Expanded spectrum	174	BSI	MRSA
67	M	HM2	Narrow spectrum	175	BSI	*Streptococcus bovis*
63	M	HVAD	Narrow spectrum	177	PCI, PK, DL, MN	MSSA
69	M	HVAD	Narrow spectrum	184	PCI	MSSA
61	M	HVAD	Narrow spectrum	190	BSI, PCI, DL	MSSA
46	M	HM2	Narrow spectrum	210	BSI, PCI, DL	MSSA
69	M	HVAD	Narrow spectrum	218	BSI	*Enterococcus* spp
66	M	HM3	Expanded spectrum	229	DL	MSSA
78	M	HM2	Narrow spectrum	230	Pocket	CONS, *C acnes*
54	M	HM2	Narrow spectrum	249	DL	MRSA
69	M	HM2	Narrow spectrum	254	DL	MSSA
35	M	HM2	Narrow spectrum	278	DL	*Neisseria sicca*
60	F	HVAD	Narrow spectrum	311	BSI, PCI	MRSA
62	M	HM2	Narrow spectrum	358	PK	MSSA

Abbreviations: BSI, bloodstream infection; CONS, coagulase-negative staphylococci; DL, driveline infection; F, female; HA-5, Heart assist 5; HM2, Heartmate 2; HM3, Heartmate 3; HVAD, HeartWare Left Ventricular Assist Devices; IE, infective endocarditis; LVAD, left ventricular assistant device; LVADI, LVAD infection; M, male; MN, mediastinitis; MRSA, methicillin-resistant *Staphylococcus aureus*; MSSA, methicillin-susceptible *S aureus*; PCI, pump or cannula infection; PK, pocket infection; PSA, *Pseudomonas aeruginosa*; SIP, surgical infection prophylaxis; VRE, vancomycin-resistant enterococci.

Overall, there were 272 deaths during a median follow up of 9.6 years (IQR, 6.6–12.7), corresponding to a 1-, 5-, and 10-year cumulative mortality rates of 21.1%, 52.8%, and 73.4%. After an episode of LVADI, 26 patients died over a median follow up of 7.1 years (IQR, 5.0–9.1); 3 patients died within 1 month of the LVADI (organisms were MSSA BSI, *Streptococcus bovis* BSI, and *Klebsiella* spp BSI). Using time-dependent Cox regression analysis, the development of LVADI did not significantly affect overall survival (*P* = .670). In this multivariable model, older age and increasing Charlson index score were associated with worse overall survival (both *P* < .001) (Table 5).

**Table 5.** Association of LVAD Infections on Survival

Predictor	Comparison	Hazard Ratio (95% Confidence Interval)	*P* Value
LVAD infection	Yes/No	1.10 (.72–1.67)	.670
Age at time of LVAD	69.2 years:53.2 years	2.12 (1.47–3.04)	<.001
Sex	Female/Male	.96 (.69–1.34)	.832
Year of LVAD	2015.5:2010.4	1.08 (.86–1.35)	.793
Indication for LVAD	Bridge/Destination	1.01 (.78–1.30)	.951
MRSA colonization	Yes/No	1.31 (.86–2.01)	.214
VRE colonization	Yes/No	1.22 (.81–1.82)	.337
Charlson comorbidity index	8:4	1.52 (1.23–1.88)	<.001
Antimicrobial prophylaxis	Expanded spectrum/Narrow spectrum	1.11 (.83–1.47)	.487

Abbreviations: LVAD, left ventricular assistant device; MRSA, methicillin-resistant *Staphylococcus aureus*; VRE, vancomycin-resistant enterococci.

Note: Results were obtained from a multivariable Cox PH regression model for time to death, with LVAD infection incorporated in the model as a time-dependent covariate. For each continuous covariate, we estimated the hazard ratio associated with the variable changing from its first to third quartile; all other covariates held constant.

## DISCUSSION

This is a single-center, retrospective cohort study evaluating antimicrobial prophylaxis strategies, incidence of LVADI, CDI, and mortality up to 12 months after LVAD placement. In our unadjusted results, we report evidence supporting noninferiority, and even superiority, of the narrow-spectrum over expanded-spectrum prophylaxis regimens with respect to LVADI. We also demonstrated noninferiority for the narrow-spectrum regimen with respect to *C difficile* infection. The most common LVADI was bloodstream infection, followed by driveline infections most frequently caused by Gram-positive bacteria.

There are multiple positions regarding an optimal LVAD placement antimicrobial prophylaxis strategy and practices vary widely [5, 8, 9]. In 2017, the ISHLT published a consensus statement highlighting that despite lack of extensive data to guide SIP, they recommend a regimen that would target *Staphylococcus* spp, including MRSA coverage if needed. Routine use of Gram-negative or fungal coverage was not recommended [10], with a similar suggestion in patients who had chest tubes at the time of surgery. Several studies have evaluated outcomes of prophylaxis after implementation of ISHLT guidelines. Furthermore, in 2023, the ISHLT published a 10-year update for the guidelines for mechanical circulatory support. In this document, they reiterate the prior recommendations in terms of Gram-positive coverage and no coverage for Gram-negative organisms. They also discuss new data in the use of vancomycin in high-risk patients such as higher body mass index, reoperation, renal failure or diabetes, and immunosuppressed patients [11], which will need to be assessed in other studies.

In 2018, Aburjania et al [12] evaluated 239 patients who underwent LVAD placement and did not find any difference in incidence of infection among the 2 groups (multidrug vs single SIP). In 2022, Allen et al [13] conducted a retrospective review of patients who received 4-drug versus single SIP. Their findings were consistent with our study demonstrating a lower rate of LVADI in the single-drug regimen compared to the multidrug regimen as well as no difference in the incidence of Gram-negative or fungal infections between the 2 groups [13]. Nguyen et al [14] also performed a similar study analyzing incident infection and mortality at 1 and 12 months of LVAD implantation with no difference noted between narrow versus broad prophylaxis. Few meta-analyses have shown similar findings in terms of LVADI incidence, with 1 study reporting a lower survival rate in patients who received a multidrug SIP [15], confirming again that there is likely no advantage of multidrug prophylaxis.

Length of stay and ICU stay are both potential confounders, but adjustment for these did not change our results. In terms of adverse drug effects, it is well known that exposure to broad-spectrum antibiotics increases the risk of antibiotic resistance. Farmakiotis et al [16] performed an analysis of pathogen susceptibilities during the first episodes of infections after LVAD placement, and they found that there was an increased rate of resistance to tetracyclines and trimethoprim-sulfamethoxazole in patients who had prolonged oral antibiotics to prevent the incidence of LVADI. Even though this is not strictly related to surgical prophylaxis, this highlights that the unneeded exposure to antibiotics increases the risk of resistance [16]. This is of particular importance in this population because they can later present with LVADI that require multiple antibiotic therapies, and, in severe cases, some patients may even need suppression therapy. Thus, preserving the availability of oral antibiotic options is crucial. On the other hand, some patients may eventually receive a heart transplant. In these patients, given the inevitable nature of chronic immunosuppression, minimizing the risk of resistance and preserving all possible antimicrobial options is of equal importance. On the same line, it is important to highlight that LVADI can result in postcardiac transplantation outcomes, including mortality [17].

It must be noted that the main goal of surgical prophylaxis is to prevent a surgical site infection. However, the risks surrounding LVADI within an early postsurgical period involve more than the surgical site alone, because the device's driveline is exposed to the environment. Some infections related to LVAD such as BSI cannot be really controlled by any surgical prophylaxis, but it can affect the LVAD integrity and cause LVADI as well. Therefore, we need to understand that implantation prophylaxis can do so much more to prevent these complications, and there is no benefit of exposing the patients to extensive regimens that may have more than harm than benefit.

With regards to not finding a clear difference in LVADI risk within the first 1.5 months postimplantation between regimens, a plausible explanation could be that certain risk features may evolve and/or influence risk differentially over time. In contrast, we did find that expanded-spectrum prophylaxis was associated with a higher number of LVADI after 3, 6, and 12 months; unfortunately, in our cohort, we were unable to identify features that would explain this higher risk. In a complex patient population with a 12-month follow up, many variables could factor into shifting evolution and contribution of risk over time. As a retrospective, observational study, it is methodologically challenging to account for every such variable to account for which one may have driven the differences across the follow-up period; however, we find the most pertinent and useful finding is that we did not observe narrow-spectrum prophylaxis regimens to be associated with a greater risk of LVADI in either the early or late postimplantation phase.

To our knowledge, this is the largest published cohort of patients who underwent LVAD implantation, including 399 patients in a timeframe of 12 years. The benefit of collecting data in such a long timeframe is that we can evaluate the different types of LVAD because it is well known that the newer devices are usually easier to implant, smaller, and have fewer complications. This timeframe also allowed us to evaluate impacts of the evolution of prophylaxis practices after emergence of early evidence, suggesting that using narrow-spectrum regimens is adequate. Another strength is that we were able to rule out group differences due to certain baseline characteristics and potential risk factors for nosocomial infections, such as length of stay and line or urinary catheter placement. To our knowledge, this is the first study that also takes these into account these variables.

The main limitation of our investigation involves the retrospective nature of this study and the potential for selection bias with observational treatment comparisons. No clear treatment selection bias was evident with respect to the descriptors collected and displayed in Tables 1 and 2, suggesting that the 2 prophylaxis groups are potentially comparable at baseline. However, we cannot exclude the possibility of important clinical variables related to risk for LVADI that may have been missing from our data collection and could have impacted the results of our outcome analyses. Furthermore, the small number of events for each outcome limited the feasibility of multivariable regression adjustment. Therefore, due to the lack of randomization and statistical adjustments, these unadjusted group comparisons are susceptible to confounding and should be interpreted with some caution. Another limitation is that given the timeframe of the study, our data do not reflect the latest types of LVAD, such as HeartMate-3.

## CONCLUSIONS

In conclusion, these unadjusted comparisons among LVAD recipients showed evidence supporting noninferiority, and even superiority, of the narrow-spectrum over expanded-spectrum prophylactic regimens with respect to risk of LVAD infection through 12 months. Although we were not able to establish superiority with respect to 12-month risk of *C difficile* infection, narrow-spectrum regimens fared at least as well as the expanded-spectrum group. Considering the risks associated with broad-spectrum antibiotic exposure, as well as the current evidence behind it, our data show that the current recommendation of ISHLT for single-drug surgical prophylaxis is a safe strategy in patients undergoing LVAD placement. Further prospective studies are needed to confirm these findings, especially with the increasing demand of advanced mechanical heart failure support.
